# Dual function of auxin during leaf abscission in poplar

**DOI:** 10.1186/1753-6561-5-S7-O26

**Published:** 2011-09-13

**Authors:** Xu Jin, Urs Fischer

**Affiliations:** 1 Swedish Agricultural University, Umeå Plant Science Centre, Department of Forest Genetics and Plant Physiology, Umeå, Sweden; 2 University of Göttingen, Forest Botany and Tree Physiology, Göttingen, Germany

## Background

Leaf abscission is an important trait for biomass production and seasonal acclimation in deciduous trees of the temperate region. Various plant hormones are involved in the timing of abscission. For example, ethylene signaling is required to induce hydrolysis of cell walls, while an auxin gradient [1] was suggested to act upstream of ethylene on the onset of leaf abscission. Besides pharmacological application of auxins on cut surfaces of explants, experimental evidence for such a gradient is however lacking. In addition to its function in temporal control, auxin has also been suggested to be a positional signal specifying the cells of the abscission zone [2].

## Methods

We established an experimental system on intact Populus trees, which allows us to induce abscission synchronously under controlled conditions. Leaf blades were bagged in aluminum foil and abscission was recorded daily. Cumulative abscission followed a sigmoidal curve for dark-induced leaves, whereas control leaves in transparent bags of the same weight as the aluminum foil bags were not separated from the stem. Abscission was preceded by senescence in the petiole but not in the leaf.

## Results and conclusions

Local auxin applications directly onto the abscission zone, as well as onto the distal end of the petiole, delayed dark induced abscission indicating that auxin could range not only as a short but also as a long distance signal. Similarly, an inhibitor of polar auxin transport retarded separation from the plant body. By contrast, auxin applied onto mature abscission zones only delayed abscission slightly in comparison to auxin applications before the development of an abscission zone. Taken together this points to a distinct function of auxin in early stages of abscission. Interestingly, we found shortly after dark-induction a new auxin response maximum on the abaxial side of the petiole, highlighting the incipient abscission zone. This auxin response maximum progressively moved from the abaxial to the adaxial side of the petiole, preceding the maturation of the abscission zone, presumably providing positional information for the formation of the abscission zone. Microarray data identified the auxin efflux carriers *PIN1* and *PIN5*, as well as a novel auxin transporter, to be down-regulated after dark induction. Immunolocalizations of those carriers will reveal if their subcellular localization and expression can explain the novel auxin response maximum.
